# Long-term efficacy and cost-effectiveness of blended cognitive behavior therapy for high fear of recurrence in breast, prostate and colorectal Cancer survivors: follow-up of the SWORD randomized controlled trial

**DOI:** 10.1186/s12885-019-5615-3

**Published:** 2019-05-16

**Authors:** Rens Burm, Belinda Thewes, Laura Rodwell, Wietske Kievit, Anne Speckens, Marieke van de Wal, Judith Prins

**Affiliations:** 1 Department of Medical Psychology, Radboud university medical center, Radboud Institute for Health Sciences, Nijmegen, The Netherlands; 2 MedValue, PO Box 9101, 6500 HB Nijmegen, The Netherlands; 3 Department for Health Evidence, Radboud university medical center, Radboud Institute for Health Sciences, Nijmegen, The Netherlands; 4 Department of Psychiatry, Radboud university medical center, Radboud Institute for Health Sciences, Nijmegen, The Netherlands; 5 Maxima Medical Center, Department of Medical Psychology, Eindhoven/Veldhoven, The Netherlands

**Keywords:** Psycho-oncology, Survivorship, Fear of cancer recurrence, Blended cognitive behaviour therapy, Cost-effectiveness, Randomized controlled trial

## Abstract

**Background:**

Blended cognitive behaviour therapy (bCBT) is an effective treatment for fear of cancer recurrence (FCR) in curatively-treated breast, colorectal and prostate cancer survivors with high FCR. However, long-term outcomes are unknown. This study investigated the long-term efficacy and cost-effectiveness of bCBT compared with care as usual (CAU).

**Methods:**

Eighty-eight cancer survivors with high FCR (Cancer Worry Scale ≥14) were randomly assigned to bCBT (*n* = 45) or CAU (*n* = 43). Data were collected at baseline and at three, nine and fifteen months from baseline and analysed by modified intention-to-treat. Efficacy was investigated with linear mixed-effects models. Cost-effectiveness was investigated from a societal perspective by comparing costs with quality-adjusted life-years (QALYs).

**Results:**

Participants who received bCBT reported significantly lower FCR compared with CAU (mean difference of − 1.787 [95% CI -3.251 to − 0.323, *p* = 0.017] at 15 months follow-up), and proportionally greater self-rated and clinically significant improvement at each follow-up measurement. Total QALYs were non-significantly different between conditions when adjusted for utility score baseline differences (0.984 compared to 0.957, *p* = 0.385), while total costs were €631 lower (95% CI -1737 to 2794, *p* = 0.587). Intervention costs of bCBT were €466. The incremental cost-effectiveness ratio amounted to an additional €2049 per QALY gained, with a 62% probability that bCBT is cost-effective at a willingness to pay (WTP) threshold of €20,000 per QALY. Results were confirmed in sensitivity analyses.

**Conclusions:**

bCBT for cancer survivors with FCR is clinically and statistically more effective than CAU on the long-term. In addition, bCBT is a relatively inexpensive intervention with similar costs and QALYs as CAU.

**Trial registration:**

The RCT was registered in the Dutch National Trial Register (NTR4423) on 12-Feb-2014. This abstract was previously presented at the International Psycho-Oncology Society conference of 2018 and published online. (Psycho-oncology, 27(S3):8-55; 2018)

**Electronic supplementary material:**

The online version of this article (10.1186/s12885-019-5615-3) contains supplementary material, which is available to authorized users.

## Background

Fear of cancer recurrence (FCR), the “fear, worry, or concern relating to the possibility that cancer will come back or progress” [1, 2], is highly prevalent amongst cancer survivors. Although some degree of FCR is normal and adaptive, high levels are associated with reduced quality of life, functional impairment, distress, anxiety, depression, excessive reassurance-seeking and increased healthcare costs [3–8]. It is estimated that around a third (31–38%) of Dutch curatively-treated cancer survivors experience high FCR [3, 9–11]. FCR seems to be a universal concern of cancer survivors rather than a cancer type-specific problem, estimates of FCR did not differ by cancer types [12]. There is consistent evidence that younger age, presence and severity of physical symptoms, psychological distress, lower quality of life, female gender and lower education are associated with higher FCR [3].

Four randomised controlled trials (RCTs) of therapist-delivered interventions to help cancer survivors manage FCR have been published to date [13–16]. Although the theoretical foundations, formats and delivery methods of these interventions differ, all available treatments are based on cognitive behaviour therapy (CBT) and some are delivered online via the internet (eHealth) [14, 17, 18]. Online CBT is a promising and potentially cost-effective treatment modality [19–21], which is at least as effective as face-to-face CBT for many mental disorders [22–25]. Online CBT may reduce waiting lists, travel time and costs [26], productivity losses [20, 26], and improve accessibility [20]. Possible disadvantages of online therapy include a lack of personal interaction, poorer adherence and less engagement [19]. Blended therapy, in which face-to-face and online therapy are combined, might bridge this gap. The Survivors’ Worries of Recurrent Disease (SWORD) study is a blended CBT (bCBT) treatment for high FCR. Immediate post-treatment efficacy has been established in an RCT which compared bCBT with care as usual (CAU) in Dutch cancer survivors with high FCR [14]. FCR was significantly reduced at 3 months follow-up in participants who received bCBT compared to those who received CAU (Cancer Worry Scale mean difference of − 3.48, 95% CI -4.69 to − 2.28, *p* < 0.001) with a medium to large effect size (d = 0.76) [14]. More detailed methodology and results are reported elsewhere [14, 27]. Efficacy at 6 and 11 months post-treatment has been demonstrated for two other FCR interventions [13, 16], however efficacy beyond the first year after treatment has received little attention. Only one trial to date has included a follow-up beyond the first year after treatment ended; this study of a therapist-delivered FCR intervention found that treatment was not more effective than CAU in reducing FCR at 15 months [15]. No previous studies have evaluated the cost-effectiveness of an individual psychological intervention for FCR, however one study has evaluated the cost-effectiveness of two group-based interventions to reduce fear of progression in cancer patients of mixed disease stage [28]. This study found that group CBT was less costly than supportive-experiential group therapy with a similar reduction of fear of progression [28].

With increasingly constrained healthcare budgets it is important to ensure that the benefits of an intervention outweigh the costs [29]. Therefore, this study aimed to evaluate the efficacy and cost-effectiveness of the SWORD intervention at 9 and 15 months follow-up.

## Methods

### Study design and participants

Detailed information concerning study design, recruitment and inclusion/exclusion criteria is published elsewhere [27]. Between 2014 and 2016, a multicentre prospective two-arm RCT was conducted investigating the efficacy of bCBT in cancer survivors with high FCR (Cancer Worry Scale score ≥ 14). In total, 88 curatively-treated breast (BC), prostate (PC) and colorectal cancer (CRC) survivors who had completed primary medical treatment at least six months and no longer than five years were included. Eligible participants who gave informed consent were randomly assigned to receive either CAU or bCBT. The RCT was approved by an ethical board (CMO Arnhem-Nijmegen), registered in the Dutch National Trial Register (NTR4423) and adheres to CONSORT guidelines.

### Intervention and care as usual

The intervention was delivered as blended care during a 3-month period: five individual one-hour face-to-face sessions (sessions 1, 2, 3, 5, 8) combined with three 15-min e-consultations (chat application, sessions 4, 6, 7) and access to a website. Therapeutic techniques applied in the intervention included psycho-education, cognitive restructuring, and behavioral modification. One face-to-face booster session followed at three months post-therapy. Participants who received CAU had no restrictions regarding the use of other psychosocial support during the study period.

The website was developed in agreement with Dutch privacy laws equal to HIPAA. All communication from and to their servers was encrypted. The hosting network was certified.

### Outcome measures

Questionnaires were completed before randomization (baseline, T0), and at three months (T1), nine months (T2) and fifteen months (T3) after baseline assessment. The primary outcome measure was FCR, assessed with the Cancer Worry Scale (CWS). The CWS is a reliable and valid questionnaire which measures FCR on an 8-item 4-level scale [10]. Total scores range between 8 and 32 and a cut-off score of ≤13 versus ≥14 differentiates between low and high FCR [10]. Secondary outcomes were FCR severity (Fear of Cancer Recurrence Inventory [FCRI] severity subscale) [30], cancer-specific distress (Impact of Events Scale [IES]) [31, 32], distress (Hospital Anxiety and Depression Scale [HADS]) [33], fatigue (Checklist Individual Strength 8R [CIS-8R]) [34], and health-related quality of life (HRQoL; European Organisation for Research and Treatment of Cancer Quality of Life Questionnaire C30 [EORTC QLQ-C30]) [35].

Costs and quality-adjusted life years (QALYs) were used as the outcome measures in the cost-effectiveness analyses. The QALY combines quality (weighed by utility scores) and quantity of life (life years gained) in one measure and is the predominant outcome measure used in health economic evaluations to assess value for money of interventions. QALYs enable comparison of cost-effectiveness of different interventions across different disease areas. Each QALY gained is worth a certain maximum monetary value that governmental organizations or insurance companies are willing to pay; also called willingness to pay (WTP) thresholds. To calculate QALYs, utility scores were obtained by using the EuroQol five dimensions three-level questionnaire (EQ-5D-3 L) and by applying the Dutch EQ-5D tariff [36, 37]. Utility scores are preference-based quality of life values, generally ranging from 0 (death) to 1 (perfect health). These utilities were multiplied by the duration of follow-up (fifteen months) to calculate QALYs.

The economic evaluation was conducted from a societal perspective, and therefore included medical costs (healthcare resource use and medication), non-medical costs (informal care and loss of productivity from paid work) and costs of the bCBT intervention. Both medical- and non-medical resource use were measured by pre-defined self-reported cost diaries collected at T1, T2 and T3. Resource use volumes were valued using standard Dutch reference prices for 2014 if available (Additional file 1: Appendix 1) [29]. The economic impact of absence from paid work was calculated using the friction cost method [38], which was set at 12 weeks in 2014 in the Netherlands [29]. Intervention program costs included bottom-up calculated costs of psychologist training, supervision and consultations, in addition to costs of website development, updates and user licences divided by a conservative amount of potential annual users in a regional implementation scenario (Additional file 1: Appendix 2) [39, 40]. No costs or effects were discounted due to the short study period.

### Statistical analysis

For the analysis of the primary outcome of FCR, all secondary outcomes and health-economic outcomes, a modified intention-to-treat (mITT) analysis was performed; in which participants who experienced a recurrence of cancer during the follow-up period were retained in the analysis, but only their scores before recurrence were included in the analysis.

#### Clinical outcomes

Long-term effects of bCBT on primary and secondary outcomes were examined by fitting a linear mixed-effects model for mean CWS follow-up scores. This model included treatment condition, follow-up time (using discrete time points) and their interaction, with an unstructured residual-error covariance matrix specified to account for the within-person correlation on the repeated follow-up measures. Baseline score and primary cancer type were included as covariates. The latter was a stratification variable for randomisation and was also found to be associated with the probability of missing data at T2 and T3. For the primary outcome of FCR, two sensitivity analyses were conducted. In the first sensitivity analysis, participants who reported a recurrence were excluded from the analysis dataset. The second sensitivity analysis was a per protocol analysis of participants who completed the bCBT intervention (intervention arm) and who had full follow-up data on FCR. All analyses for the clinical outcomes were conducted in Stata 14.2.

Clinically significant improvement (CSI) on levels of FCR between baseline and both T2 and T3 follow-up assessments was established by combining statistically reliable improvement (reliable change index < 1.96) [41] and a decrease of CWS scores to the normal range (clinically significant change, CWS < 14) [27]. Self-rated improvement had occurred if the participant answered ‘yes’ to ‘feeling much better but still experiencing some FCR’ or ‘no longer experiencing FCR’.

#### Health economic outcomes

Missing total cost and utility score data per measurement were imputed at the aggregated level separately per treatment arm, by using multiple imputation (MI) by chained equations with predictive mean-matching [42, 43]. Twenty imputed datasets were created and pooled according to Rubin’s rules [44].

First of all, incremental costs and incremental QALYs between the CAU and bCBT strategies were calculated and statistical differences in non-normally distributed costs and QALYs were tested with bootstrapped t-tests [45]. Non-parametric percentile bootstrapping with 1000 replications was performed to estimate 95% confidence intervals [46]. In order to weigh incremental costs against incremental QALYs, the incremental cost-effectiveness ratio (ICER) was calculated by dividing total incremental costs by the incremental QALYs to obtain the costs per QALY gained by the intervention [39]. Due to substantial differences in EQ-5D baseline utility scores between conditions, regression-based adjustment was performed within the net monetary benefit framework, which values both benefits and costs in monetary terms and therefore enables the use of regression methods [47]. Incremental net monetary benefit (INMB) statistics were estimated for different WTP thresholds. The INMB was calculated by using the following formula: (incremental QALY * WTP) – incremental costs. Cost-effectiveness acceptability curves (CEACs) were constructed which display the probability that bCBT would be cost-effective given specific WTP thresholds [48]. A sensitivity analysis was conducted which used condition-specific utility scores calculated from the EORTC QLQ-C30 instead of utility scores calculated from the EQ-5D-3 L [49].

## Results

### Sample characteristics

**Fig. 1:**
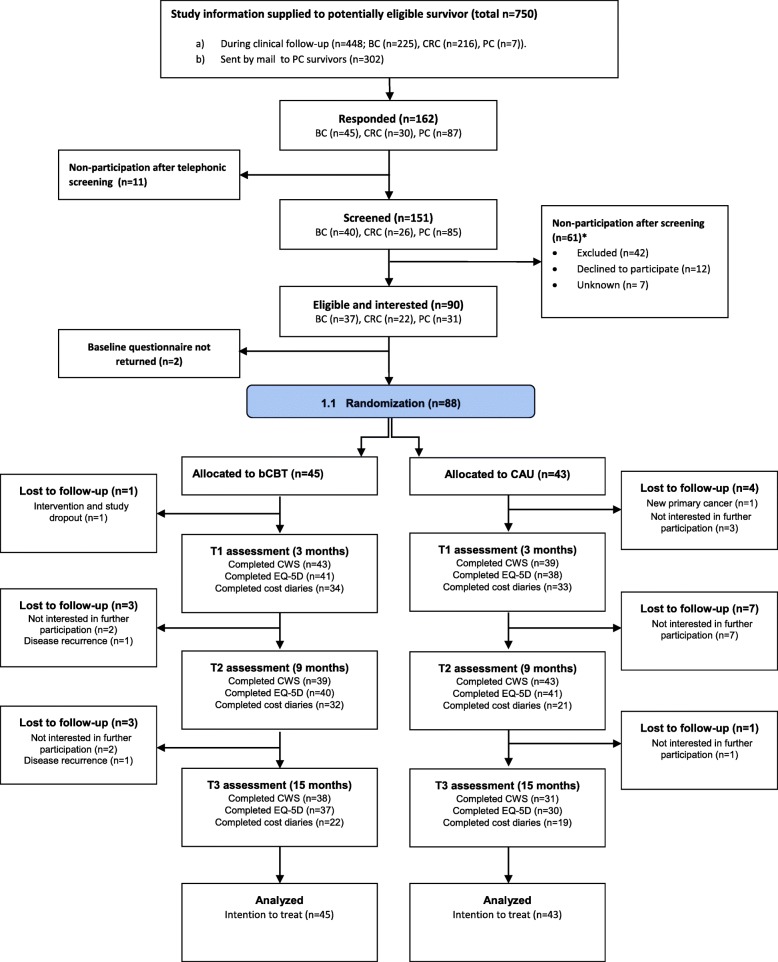
CONSORT flow diagram showing recruitment and enrolment of 88 participants. Participants who reported a recurrence, but still filled in questionnaires while they had a recurrence (6 additional patients in the bCBT group), are not listed as ‘lost to follow up’. Abbreviations: bCBT, blended cognitive behaviour therapy; CAU, care as usual; BC, breast cancer; CRC, colorectal cancer; PC, prostate cancer; CWS, cancer worry scale; EQ-5D, EuroQol five dimensions questionnaire

In total, 45 participants were randomly assigned to bCBT and 43 to CAU (Fig. 1). Both conditions were comparable on demographic characteristics and clinical characteristics (Table 1). Nine participants reported a recurrence at some point during follow-up (bCBT: 8, CAU: 1) and one participant receiving CAU reported a new primary cancer. Three of these patients dropped out of the study (bCBT: 2, CAU: 1). Furthermore, 16 participants dropped out of the study during follow-up for various other reasons (bCBT: 5, CAU: 11; Fig. 1). Having had colorectal cancer (*p* = 0.047) and having comorbid conditions (*p* = 0.044) was significantly associated with dropout at any stage during the study. The number of completers (who completed all CWS questionnaires and, for bCBT, the full intervention) was 23 in the bCBT and 30 in the CAU condition.

**Table 1:** Demographic and clinical characteristics at baseline of participants by study group

	bCBT (*n* = 45)	CAU (*n* = 43)
Characteristic	*n* (%)	*n* (%)
Age		
Mean (SD)	58.0 (11.3)	59.7 (10.0)
Gender		
Male	21 (46.7%)	20 (46.5%)
Education		
Low (ISCED 0–2)	11 (24.4%)	18 (41.9%)
Middle (ISCED 3–5)	19 (42.2%)	10 (23.3%)
High (ISCED 6–8)	15 (33.3%)	15 (34.9%)
Employment status		
Paid Employment	17 (37.8%)	17 (39.5%)
Unemployed	8 (17.8%)	5 (11.6%)
Voluntary work	4 (8.9%)	8 (18.6%)
Retired	16 (35.6%)	14 (32.6%)
Primary cancer site		
Breast	18 (40.0%)	18 (41.9%)
Colorectal	12 (26.7%)	10 (23.3%)
Prostate	15 (33.3%)	15 (34.9%)
Treatment type		
Surgery	18 (40.0%)	11 (25.6%)
Radiotherapy	0 (0.0%)	3 (7.0%)
Surgery + Radiotherapy	9 (20.0%)	9 (20.9%)
Surgery + Chemotherapy	12 (26.7%)	9 (20.9%)
Surgery + Radiotherapy + Chemotherapy	6 (13.3%)	11 (25.6%)
Time since diagnosis (years)		
Mean (SD)	2.4 (1.5)	2.8 (1.3)
Time since last treatment (years)		
Mean (SD)	1.9 (1.5)	2.1 (1.4)
Number of comorbid diseases		
0	19 (42.2%)	14 (32.6%)
1–2	19 (42.2%)	22 (51.2%)
3+	7 (15.6%)	7 (16.3%)
Psychological help in the past		
yes	22 (50.0%)	26 (60.5%)

Abbreviations: *bCBT* blended cognitive behaviour therapy, *CAU* care as usual, *ISCED* International Standard Classification of Education, *SD* standard deviation

### Clinical outcomes

Results for the primary and secondary clinical outcomes are presented in Table 2. The decrease in FCR was significantly greater in participants who received bCBT compared with CAU at all follow-up measurements (Additional file 1: Appendix 3). At T3, the detected mean difference in FCR remained statistically significant (− 1.787, 95% CI -3.251 to − 0.323, *p* = 0.017), although smaller than at T1 (− 3.534, 95% CI -4.764 to − 2.305, *p* = 0.000) and T2 (− 4.104, 95% CI -5.531 to − 2.677, *p* = 0.000). Similar results were found in the per protocol sensitivity analysis. In the sensitivity analysis which excluded participants with recurrences, the reduction in FCR in the bCBT condition was larger at each follow-up measurement than in the primary analysis (Additional file 1: Appendix 4).

**Table 2:** Effect of treatment (modified intention-to-treat) on primary and secondary outcomes (*n* = 88)

	bCBT (*n* = 45)		CAU (*n* = 43)				
	Mean	SE/SD*	Mean	SE/SD*	Mean difference	95% CI	*p* value
Primary outcome							
FCR (CWS)							
T0	19.622	3.737	19.558	3.744			
T1 unadjusted	14.814	4.579	18.487	3.986			
T1 adjusted	14.743	0.432	18.277	0.454	−3.534	−4.764 to −2.305	**0.000**
T2 unadjusted	13.718	4.472	17.844	3.836			
T2 adjusted	13.916	0.493	18.020	0.534	−4.104	−5.531 to − 2.677	**0.000**
T3 unadjusted	14.684	4.539	16.516	4.081			
T3 adjusted	14.783	0.504	16.570	0.549	−1.787	−3.251 to −0.323	**0.017**
Secondary outcomes							
Multidimensional aspects of FCR							
Severity (FCRI)							
T0	21.909	4.850	23.116	6.456			
T1 unadjusted	16.605	7.251	22.282	5.991			
T1 adjusted	16.989	0.756	21.330	0.788	−4.341	−6.497 to −2.185	**0.000**
T2 unadjusted	15.025	6.897	21.313	5.171			
T2 adjusted	15.957	0.688	20.462	0.742	−4.505	−6.513 to −2.497	**0.000**
T3 unadjusted	15.842	7.343	19.733	5.539			
T3 adjusted	16.599	0.805	19.023	0.879	−2.423	−4.785 to −0.062	**0.044**
Distress, depression, anxiety							
Distress (HADS)							
T0	14.089	7.836	15.209	8.911			
T1 unadjusted	9.951	8.402	17.131	9.519			
T1 adjusted	10.749	0.731	16.003	0.763	−5.254	−7.334 to −3.174	**0.000**
T2 unadjusted	9.718	7.412	15.862	9.771			
T2 adjusted	11.139	0.806	14.897	0.898	−3.759	−6.134 to −1.383	**0.002**
T3 unadjusted	9.778	8.292	14.889	9.390			
T3 adjusted	11.414	0.966	13.228	1.082	−1.813	−4.669 to 1.043	0.213
Anxiety (HADS)							
T0	8.156	4.101	8.395	4.914			
T1 unadjusted	5.317	4.239	9.447	5.012			
T1 adjusted	5.639	0.437	8.951	0.456	−3.312	−4.555 to −2.068	**0.000**
T2 unadjusted	5.333	4.132	8.862	5.585			
T2 adjusted	6.030	0.528	8.549	0.588	−2.519	−4.074 to −0.964	**0.002**
T3 unadjusted	4.944	4.056	8.333	4.844			
T3 adjusted	5.751	0.570	7.734	0.638	−1.984	−3.667 to − 0.301	**0.021**
Depression (HADS)							
T0	5.933	4.218	6.814	4.722			
T1 unadjusted	4.634	4.598	7.684	5.152			
T1 adjusted	5.050	0.424	7.131	0.442	−2.081	−3.286 to −0.876	**0.001**
T2 unadjusted	4.385	4.017	7.000	4.751			
T2 adjusted	5.042	0.418	6.406	0.467	−1.363	−2.597 to −0.130	**0.030**
T3 unadjusted	4.833	4.675	6.556	4.956			
T3 adjusted	5.582	0.504	5.611	0.565	−.029	−1.520 to 1.462	0.970
Cancer-specific distress (IES)							
T0	23.044	14.555	24.902	15.604			
T1 unadjusted	14.732	14.998	26.026	17.747			
T1 adjusted	15.539	2.064	24.598	2.208	−9.059	−15.003 to −3.115	**0.003**
T2 unadjusted	9.973	13.710	16.643	15.905			
T2 adjusted	11.127	2.085	17.107	2.391	−5.979	−12.215 to 0.256	0.060
T3 unadjusted	14.944	14.633	18.259	15.294			
T3 adjusted	15.781	2.147	17.254	2.450	−1.473	−7.883 to 4.938	0.653
Fatigue (CIS-8R)							
T0	30.222	12.951	36.116	12.585			
T1 unadjusted	25.250	11.535	36.684	13.001			
T1 adjusted	27.961	1.137	33.478	1.176	−5.516	−8.771 to −2.262	**0.001**
T2 unadjusted	26.769	11.855	32.586	12.290			
T2 adjusted	28.694	1.297	30.512	1.444	−1.818	−5.658 to 2.022	0.353
T3 unadjusted	26.806	11.436	30.556	13.042			
T3 adjusted	29.387	1.350	26.888	1.514	2.499	−1.522 to 6.521	0.223
Quality of life (EORTC QLQ-C30)							
Global quality of life							
T0	62.222	19.672	61.434	20.252			
T1 unadjusted	73.062	19.402	57.479	20.483			
T1 adjusted	72.504	2.556	58.075	2.682	14.429	7.153 to 21.705	**0.000**
T2 unadjusted	72.083	21.229	62.500	21.792			
T2 adjusted	71.918	3.004	62.632	3.264	9.286	0.576 to 17.997	**0.037**
T3 unadjusted	73.026	19.609	65.278	19.948			
T3 adjusted	72.672	2.823	67.742	3.108	4.930	−3.331 to 13.192	0.242
Physical functioning							
T0	87.704	13.406	83.953	16.269			
T1 unadjusted	89.380	11.735	80.855	20.010			
T1 adjusted	87.552	1.527	82.886	1.606	4.666	.296 to 9.037	**0.036**
T2 unadjusted	88.750	15.148	86.458	14.809			
T2 adjusted	86.246	1.737	87.766	1.893	−1.519	−6.583 to 3.545	0.557
T3 unadjusted	89.649	13.924	84.301	14.560			
T3 adjusted	87.986	1.592	86.829	1.721	1.156	−3.479 to 5.792	0.625
Role functioning							
T0	77.037	26.181	74.806	25.295			
T1 unadjusted	85.659	21.076	74.123	23.474			
T1 adjusted	84.543	2.963	75.547	3.142	8.996	0.503 to 17.489	**0.038**
T2 unadjusted	83.750	23.415	82.292	21.972			
T2 adjusted	82.766	3.407	82.705	3.742	0.061	−9.887 to 10.008	0.991
T3 unadjusted	89.035	17.013	84.409	23.148			
T3 adjusted	87.388	2.777	85.357	3.043	2.031	−6.095 to 10.157	0.624
Emotional functioning							
T0	64.815	22.883	66.860	22.456			
T1 unadjusted	81.589	23.185	60.256	29.830			
T1 adjusted	81.647	2.934	59.978	3.078	21.669	13.326 to 30.011	**0.000**
T2 unadjusted	80.208	22.463	61.719	28.544			
T2 adjusted	78.959	3.259	60.785	3.540	18.174	8.731 to 27.617	**0.000**
T3 unadjusted	77.851	23.592	68.548	25.973			
T3 adjusted	77.507	2.987	68.668	3.242	8.839	0.179 to 17.499	**0.046**
Cognitive functioning							
T0	72.963	24.692	68.992	25.350			
T1 unadjusted	80.620	19.897	62.393	28.541			
T1 adjusted	79.977	2.920	64.662	3.071	15.315	6.989 to 23.641	**0.000**
T2 unadjusted	82.500	19.954	72.917	28.945			
T2 adjusted	80.130	3.276	74.990	3.593	5.140	−4.421 to 14.701	0.292
T3 unadjusted	79.386	22.738	68.280	25.587			
T3 adjusted	76.994	3.171	71.780	3.442	5.214	−4.013 to 14.441	0.268
Social functioning							
T0	80.000	23.192	71.318	24.754			
T1 unadjusted	89.922	17.495	76.923	26.384			
T1 adjusted	87.868	2.907	79.225	3.055	8.643	0.306 to 16.979	**0.042**
T2 unadjusted	85.833	22.504	80.208	22.175			
T2 adjusted	83.302	3.052	81.127	3.310	2.176	−6.727 to 11.078	0.632
T3 unadjusted	88.158	19.723	86.022	19.292			
T3 adjusted	85.924	2.556	89.059	2.759	3.135	−10.641 to 4.370	0.413

NOTE. Linear mixed-effects models were used to calculate all *p*-values. Adjusted means were adjusted for the baseline covariates primary cancer site and baseline value of the corresponding outcome; unadjusted means were unadjusted for covariates. Boldface type indicates a statistically significant effect for adjusted means (*p* < 0.05)

*SEs are reported for adjusted means, SDs for unadjusted means

Abbreviations: *bCBT* blended cognitive behaviour therapy, *CAU* care as usual; *FCR* fear of cancer recurrence, *CWS* Cancer Worry Scale, *FCRI* Fear of Cancer Recurrence Inventory, *HADS* Hospital Anxiety and Depression Scale, *IES* Impact of Events Scale, *CIS-8R* Checklist Individual Strength 8R, *EORTC QLQ-C30* European Organisation for Research and Treatment of Cancer Quality of Life Questionnaire C30, *SD* standard deviation, *SE* standard error

Regarding the secondary outcomes, participants receiving bCBT experienced significantly greater improvement at each follow-up measurement on anxiety, emotional functioning and the FCRI severity subscale compared with CAU. Significant improvement at T1 and T2 was found on distress, depression and global quality of life. On the other six secondary outcomes (cancer-specific distress, fatigue and physical, role, social and cognitive functioning), a statistically significant effect was found at T1.

Significantly more participants receiving bCBT compared with CAU reported reliable improvement (78% vs. 19%, *p* ≤ 0.001), clinically significant change (58% vs. 9%, *p* ≤ 0.001), clinically significant improvement (51% vs. 3%, *p* ≤ 0.001) and self-rated improvement (74% vs. 41%, *p* = 0.007) at T2. At T3, only clinically significant change (50% vs. 16%, *p* = 0.038) and self-rated improvement (72% vs. 40%, *p* = 0.020) remained significantly higher in bCBT (Additional file 1: Appendix 5).

### Health economic outcomes

Participants receiving bCBT reported substantially lower costs on most medical resource categories, including medical specialists, psychosocial help and diagnostics (Additional file 1: Appendix 6). Costs of the bCBT intervention programme amounted to €466 (Additional file 1: Appendix 2), which were added to the costs made by bCBT participants. Total costs (combined medical, non-medical and bCBT intervention costs) over the complete follow-up period were non-significantly lower in those who received bCBT compared with CAU (− €164, 95% CI -2018 to 2502, *p* = 0.891).

Results of the different cost-effectiveness analyses are shown in Table 3. Baseline EQ-5D utility scores were substantially higher in the bCBT condition (0.78 [SD = 0.18]) than in CAU (0.68 [SD = 0.22]) (Additional file 1: Appendix 3). Mean QALYs unadjusted for this baseline difference were significantly higher in the bCBT condition (1.027 compared to 0.913, *p* = 0.018). However, QALYs were non-significantly different when these were adjusted for baseline EQ-5D utility scores (0.984 compared to 0.957, *p* = 0.385) and when QALYs were calculated from QLQ-C30 utility scores (1.107 compared to 1.074, *p* = 0.137).

**Table 3:** Results of the different cost-effectiveness analyses

		Unadjusted		Adjusted		QLQ-C30	
		bCBT	CAU	bCBT	CAU	bCBT	CAU
Costs	Mean	6001	6165	6001	6165	6001	6165
	95% CI	4552 to 7664	4657 to 7850	4552 to 7664	4657 to 7850	4552 to 7664	4657 to 7850
Incremental costs	Mean	−164		−164		−164	
	95% CI	−2018 to 2502		−2018 to 2502		−2018 to 2502	
QALYs	Mean	1.027	0.913	0.984	0.957	1.107	1.074
	95% CI	0.966 to 1.083	0.839 to 0.980	0.941 to 1.028	0.912 to 1.001	1.074 to 1.135	1.046 to 1.100
Incremental QALYs	Mean	0.114		0.028		0.033	
	95% CI	0.026 to 0.206*		−0.031 to 0.090		−0.012 to 0.072	
ICER		€-1081 / QALY		€2049 / QALY		€-6189 / QALY	
INMB at WTP of 0	Mean	€164		€-57		€164	
	95% CI	€-2018 to €2502		€-2709 to €2370		€-2081 to 2419	
INMB at WTP of €20,000	Mean	€2403		€498		€807	
	95% CI	€-893 to €5454		€-2514 to €3269		€-1898 to €3249	
INMB at WTP of €50,000	Mean	€5761		€1331		€1770	
	95% CI	€386 to €11,274*		€-3189 to €5773		€-1322 to €5064	

NOTE. Unadjusted and adjusted cost-effectiveness analyses used QALYs calculated from EQ-5D utilities, while the QLQ-C30 analysis used QALYs calculated from EORTC QLQ-C30 utilities. Estimates in the adjusted cost-effectiveness analysis were adjusted for baseline EQ-5D utility scores. Confidence intervals were obtained after bootstrapping with 1000 replications

Abbreviations: *bCBT* blended cognitive behaviour therapy, *CAU* care as usual, *QLQ-C30* European Organisation for Research and Treatment of Cancer Quality of Life Questionnaire C30, *ICER* incremental cost-effectiveness ratio, *INMB* incremental net monetary benefit, *WTP* willingness to pay, *QALY* quality adjusted life-year

*Significant difference between the two conditions (*p* < 0.05)

**Fig. 2:**
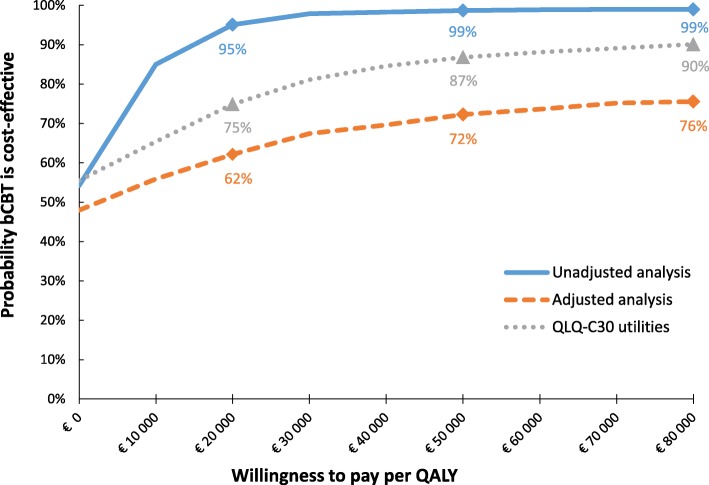
Cost-effectiveness acceptability curves of the different cost-effectiveness analyses, showing the probability that the intervention is cost-effective at different willingness to pay (WTP) thresholds. Abbreviations: bCBT, blended cognitive behaviour therapy; QALY, quality-adjusted life year; QLQ-C30, European Organisation for Research and Treatment of Cancer Quality of Life Questionnaire C30

Since the total costs were lower and total QALYs were higher when QALYs were unadjusted for baseline utility differences, bCBT was the dominant strategy (negative ICER) in this analysis. The same applies to the analysis which used QALYs calculated from QLQ-C30 utility scores instead of EQ-5D utility scores. An ICER of €2049 per QALY gained was found when QALYs were adjusted for baseline EQ-5D utility differences, since bCBT was both less costly and less effective in this analysis. INMBs in the unadjusted and QLQ-C30 analyses were in favour of the bCBT condition regardless of society’s WTP per QALY gained, while adjusted INMBs were in favour of bCBT above a WTP of €2049 per QALY gained (which is equal to the ICER). However, all INMBs had wide confidence intervals, most of which included 0 at all WTP values. The CEAC shows that there is a 48 to 56% probability that bCBT is cost-saving compared to CAU, and a 62 to 95% probability that bCBT is more cost-effective than CAU at a WTP of €20,000 (Fig. 2). This probability obviously increases as WTP values increase.

## Discussion

This study investigated the long-term efficacy and cost-effectiveness of bCBT to reduce FCR in PC, BC and CRC survivors with high FCR. It was shown previously that bCBT resulted in a statistically and clinically significant reduction of FCR severity immediately post-treatment (3 months) compared with CAU [14]. The current study found that the effect of bCBT remained significant during follow-up at 9 and 15 months [50]. Findings were confirmed in the sensitivity analyses. Furthermore, more bCBT than CAU participants reported self-rated improvement and clinically significant improvement at 9 and 15 months. However, the difference in clinically significant improvement at T3 was not statistically significant, reflecting the finding that FCR increased slightly in the bCBT condition and significantly decreased in the CAU condition between 9 and 15 months from baseline. The decrease in FCR over time in the CAU condition is inconsistent with previous literature reviews stating that high FCR remains stable over time without intervention [3, 5, 51, 52]. However, CAU participants received considerably more additional psychosocial support (by either a psychologist, mental health nurse, social worker or psychiatrist) than bCBT participants which may explain this decrease. For secondary outcomes, those receiving bCBT improved significantly on multidimensional aspects of FCR, anxiety and emotional functioning at each follow-up measurement. Other secondary outcomes (distress, depression, global quality of life) improved at T1 and T2, while the remainder (cancer-specific distress, fatigue and physical, role, social and cognitive functioning) only improved at 3 months follow-up.

In the economic evaluation, total costs were found to be numerically but not significantly lower in the bCBT condition. Intervention costs were relatively low when calculated for a conservative annual number of potential users. The amount of additional QALYs was small and not statistically significant when adjusted for baseline EQ-5D utility scores and in the sensitivity analysis using QLQ-C30 utility scores. All cost-effectiveness analyses resulted in low costs of bCBT per QALY gained and relatively high probabilities that bCBT is cost-saving compared to CAU. The maximum WTP for an additional QALY in the Netherlands is dependent on the burden of disease of the addressed condition, ranging from €20,000 to €80,000 [53]. As the disease burden of high FCR is not yet reported, we decided to use the most conservative WTP of €20,000; at which the probability that bCBT is cost effective ranged from 62 to 95%. However, since total costs and QALYs were not significantly different and the INMBs had relatively wide confidence intervals which included zero, it cannot be concluded that bCBT is more cost-effective than CAU. Instead, it could be concluded that bCBT and CAU are similar in terms of costs and QALYs. Nonetheless, as costs of eHealth interventions are partly dependant on the number of users [54], it is envisaged that when implemented in routine care, the intervention will probably become more cost-effective than demonstrated here.

Like other recent RCTs of psychological interventions, the present study has demonstrated immediate and medium-term benefits of a CBT-based psychological intervention for FCR. However, to our knowledge, this is the first study to demonstrate efficacy and cost-effectiveness of a psychological intervention for FCR beyond the first year after treatment. Several on-going trials of interventions for FCR include cost-effectiveness outcomes, and it will be interesting to compare health-economic results [55–57].

Methodological strengths of this study include the rigorous RCT design and the broad range of different clinical and health economic outcomes. Both statistical and clinical change was evaluated, and findings were adjusted for covariates and confirmed by sensitivity analyses. Despite being adequately powered, a relatively high number of dropouts (18%) and participants with recurrence (11%) was observed during follow-up. Recurrences were self-reported by participants and not systematically investigated in medical records for privacy reasons. More participants in the bCBT than in the CAU condition reported a recurrence, which may have occurred due to higher follow-up adherence of the intervention group. It is noteworthy that a number of participants with recurrence still completed follow-up questionnaires; however, their data after recurrence was excluded from the main analysis since we were only interested in the efficacy and cost-effectiveness of bCBT in disease-free cancer survivors. Although it is possible that the intervention remains suitable for people with recurrent disease, the extent to which bCBT translates to or requires adaptation for patients with advanced disease remains a question for future research.

A further limitation is that this study was not powered to detect differences in cost-effectiveness and therefore these outcomes should be interpreted with caution. Self-reported cost diaries are considered to be feasible and valid for collecting cost data [58], although in the current study it was not possible to distinguish between routine follow-up consultations and consultations due to reassurance-seeking behaviour related to FCR, which may have influenced results. Furthermore, baseline cost data, inability to perform unpaid work, limited job performance while at work and travel costs were not measured.

Baseline differences on EQ-5D utility scores were substantial while all other baseline characteristics were comparable between conditions. These differences are likely to have occurred by chance, as randomisation was performed independently by a computer-generated allocation sequence in the RCT. However, the EQ-5D has been previously reported to be less sensitive to changes in psychological well-being due to dominance of physical health domains [59]. It is possible that the psychological well-being item in the EQ-5D (‘anxiety/depression’) may not be sensitive to the psychological impact of FCR, as cancer survivors may see FCR as a normal or rational fear and not consider themselves to be anxious or depressed. Indeed, a relatively large proportion of participants in both conditions (33% in CAU and 47% in bCBT) reported no problems with anxiety or depression at baseline, even though all had high FCR at inclusion. Therefore, we additionally used the EORTC QLQ-C30 to calculate QALYs, which may be more sensitive than the EQ-5D in this population. This analysis resulted in higher mean QALY values (as expected) [49], but incremental QALYs were comparable to findings in the adjusted EQ-5D analysis; indicating that those results are robust.

## Conclusions

This study demonstrates that bCBT is statistically and clinically more effective than CAU over the first 15 months post-treatment, and a relatively inexpensive intervention with similar costs and QALYs as CAU. However, results should be replicated in larger samples and in subgroups of the investigated population.

## Additional file

Additional file 1: **Appendix 1.** Reference prices per healthcare resource category. A list of all healthcare resources measured by the cost diaries in this study and the associated unit cost per resource, which were used to calculate costs per study participant. **Appendix 2.** Calculation of bCBT intervention programme costs. A description of how bCBT intervention costs were calculated bottom-up; which were subsequently used in the cost-effectiveness analyses for the bCBT group. **Appendix 3.** Figures of mean CWS scores, mean EQ-5D utility scores and mean EORTC QLQ-C30 utility scores over time by study group. Visual representation of mean CWS scores, EQ-5D utility scores (unadjusted) and EORTC QLQ-C30 utility scores at each follow-up measurement (3, 9, 15 months). **Appendix 4.** Sensitivity analyses results conducted on the primary CWS outcome. Results of the sensitivity analysis which excluded participants who reported a recurrence and the per protocol sensitivity analysis, which only included participants who completed the intervention and who had full follow-up FCR data. **Appendix 5.** Reliable improvement, clinically significant change, clinically significant improvement and self-rated improvement in FCR severity. Results of analyses which investigated the clinical relevance of FCR improvement at 9 months and 15 months follow-up by treatment group for complete cases. Results of clinical relevancy analyses at 3 months follow-up are published elsewhere [15]. **Appendix 6.** Costs per assessment per resource category. Table of mean costs measured per resource category per follow-up assessment per study group. Costs per resource category are listed only for complete cases, since missing cost data were imputed at the aggregated (total cost) level. (DOCX 176 kb)

## Abbreviations

BC: curatively-treated breast cancer survivor
bCBT: blended cognitive behaviour therapy
CAU: Care as usual
CEAC: Cost-effectiveness acceptability curve
CRC: Curatively-treated colorectal cancer survivor
CWS: Cancer worry scale
FCR: Fear of cancer recurrence
ICER: Incremental cost-effectiveness ratio
INMB: Incremental net monetary benefit
MI: Multiple imputation
mITT: Modified intention-to-treat
PC: curatively-treated prostate cancer survivor
QALY: Quality-adjusted life-years
RCT: Randomised controlled trial
WTP: Willingness to pay
