# Supplementary material for: Long-term efficacy and cost-effectiveness of blended cognitive behavior therapy for high fear of recurrence in breast, prostate and colorectal Cancer survivors: follow-up of the SWORD randomized controlled trial
Source: BMC Cancer. 2019 May 16;19:462. doi: 10.1186/s12885-019-5615-3 (PMC6524293; doi:10.1186/s12885-019-5615-3)
Supplement: Supplementary file 1 — Appendix 1. Reference prices per healthcare resource category. A list of all healthcare resources measured by the cost diaries in this study and the associated unit cost per resource, which were used to calculate costs per study participant. Appendix 2. Calculation of bCBT intervention programme costs. A description of how bCBT intervention costs were calculated bottom-up; which were subsequently used in the cost-effectiveness analyses for the bCBT group. Appendix 3. Figures of mean CWS scores, mean EQ-5D utility scores and mean EORTC QLQ-C30 utility scores over time by study group. Visual representation of mean CWS scores, EQ-5D utility scores (unadjusted) and EORTC QLQ-C30 utility scores at each follow-up measurement (3, 9, 15 months). Appendix 4. Sensitivity analyses results conducted on the primary CWS outcome. Results of the sensitivity analysis which excluded participants who reported a recurrence and the per protocol sensitivity analysis, which only included participants who completed the intervention and who had full follow-up FCR data. Appendix 5. Reliable improvement, clinically significant change, clinically significant improvement and self-rated improvement in FCR severity. Results of analyses which investigated the clinical relevance of FCR improvement at 9 months and 15 months follow-up by treatment group for complete cases. Results of clinical relevancy analyses at 3 months follow-up are published elsewhere [15]. Appendix 6. Costs per assessment per resource category. Table of mean costs measured per resource category per follow-up assessment per study group. Costs per resource category are listed only for complete cases, since missing cost data were imputed at the aggregated (total cost) level. (DOCX 176 kb) [file 12885_2019_5615_MOESM1_ESM.docx]

**APPENDICES SWORD**

**Appendix 1.** Reference prices and sources per healthcare resource category.

|  | **Resource unit** | **Unit cost (€)** | **Source** |
| --- | --- | --- | --- |
| *Medical* |  |  |  |
| Medical specialists |  |  | Dutch Guideline for conducting economic evaluations in healthcare [[1](#_ENREF_1)] |
|  | Surgeon | 73 |  |
|  | Radiotherapist | 91 |  |
|  | Oncologist | 91 |  |
|  | Anaesthetist | 91 |  |
|  | Cardiologist | 91 |  |
|  | Gynaecologist | 91 |  |
|  | Plastic Surgeon | 91 |  |
|  | Urologist | 91 |  |
|  | Neurologist | 99 |  |
|  | Other | 91 |  |
| General practitioner |  | 33 | Dutch Guideline for conducting economic evaluations in healthcare [[1](#_ENREF_1)] |
| Psychological help |  |  | Dutch Guideline for conducting economic evaluations in healthcare [[1](#_ENREF_1)] |
|  | Social worker | 65 |  |
|  | Mental health nurse | 17 |  |
|  | Psychologist | 98 |  |
|  | Psychiatrist | 98 |  |
| Paramedical help |  |  | Dutch Guideline for conducting economic evaluations in healthcare [[1](#_ENREF_1)] |
|  | Physical therapist | 33 |  |
|  | Oedema therapist | 33 |  |
|  | Pelvic floor therapist | 33 |  |
|  | Occupational therapist | 33 |  |
|  | Other | 33 |  |
| Diagnostics |  |  | Dutch Guideline for conducting economic evaluations in healthcare [[1](#_ENREF_1)] or, if unavailable, Price List for first-line diagnostics [[2](#_ENREF_2)] |
|  | Blood test | varies |  |
|  | X-ray | varies |  |
|  | Ultrasound | varies |  |
|  | MRI-scan | varies |  |
|  | CT-scan | varies |  |
|  | PET-scan | 1149 |  |
|  | ECG | 43 |  |
|  | Colonoscopy | 352 |  |
|  | Mammogram | 91 |  |
|  | Other |  |  |
| In-hospital stay |  |  | Dutch Guideline for conducting economic evaluations in healthcare [[1](#_ENREF_1)] |
|  | Academic hospital | 642 |  |
|  | General hospital | 443 |  |
| Home care |  |  | Dutch Guideline for conducting economic evaluations in healthcare [[1](#_ENREF_1)] |
|  | Domestic help | 20 |  |
|  | Personal care | 50 |  |
|  | Nursing care | 73 |  |
|  | Treatment at home | 120 |  |
| Other |  |  |  |
|  | Medication | varies | Standard medication prices [[3](#_ENREF_3)], combined with either self-reported medication doses, or the defined daily adult dose as specified by the WHO [[4](#_ENREF_4)] |
|  | Aids and appliances at home | varies | Self-reported by participants or, if unavailable, total costs declared per user in the Netherlands as registered in GIPdatabank [[5](#_ENREF_5)] |
|  | Alternative medicine | varies | Dutch market price |
| *Non-medical* |  |  |  |
| Informal care |  | 14 | Dutch Guideline for conducting economic evaluations in healthcare [[1](#_ENREF_1)] |
| Absenteeism from paid work | | 34.75 | Friction cost method for 2014 [[1](#_ENREF_1)] |

**Appendix 2.** Calculation of bCBT intervention programme costs.

Expected amount of users

The number of potential users of the SWORD intervention is based on the estimate of the annual number of cancer patients who could be treated with SWORD in the 11 participating sites (regional hospitals, academic hospitals, psychological institutions in total). Together, these sites conservatively estimated that a mean of 22.6% of annual patient numbers could potentially be treated with SWORD, resulting in an estimated annual amount of 583 patients.

Website development and update costs

Development costs of the website in 2013 were in total €13,291.96 incl. 21% VAT. Website update costs and transfer to a new hosting platform in 2017 were €32,446.85 incl. 21% VAT. To calculate annual depreciation and interest costs, an annuity depreciation method was used [[1](#_ENREF_1)]. For this calculation, it was assumed that the platform would have a depreciation period of 10 years with no residual value after that period. The standard Dutch interest rate of 4.2% for long loans was used [[1](#_ENREF_1)]. Based on these numbers, the depreciation and interest costs amounted to €5695 annually.

Therapist user licences

It was assumed that each hospital / institution will have 2 psychologists who will work with the SWORD intervention. The hosting platform charges €33.00 per month per therapist for use of the website. There is no limitation on the amount of patients a therapist is allowed to treat with this licence. Therefore, annual user licence costs across all sites are €726 / 583 = €14.94 per patient.

Psychologist training and supervision

Psychologists of all participating sites (in total 11 x 2=22 psychologists) will be trained by two skilled therapists experienced in delivery of SWORD. Training will consist of 2 days (1 day therapy content, 0.5 day blended-care and use of website, 0.5 day train-the-trainer module, total of 20 hours) with an additional half-day (4 hours) for review and supervision approximately 6 months later. It was assumed that all trainees are qualified as health psychologists.

Salary costs for trainers / trainees were determined bottom-up. Gross salary costs were determined by using the standard health psychologist’s salary scale with a median amount of years of experience, added by one (which would be 7 + 1 = 8 years); resulting in a monthly €4200 [[1](#_ENREF_1), [6](#_ENREF_6)]. Since there are 1558 workable hours in a 36-hour work week, salary costs per hour amount to €32.35. Extra charges on top of that are 39% [[1](#_ENREF_1)]; amounting to a total gross salary cost per hour of €44.97. Therefore, total costs per psychologist trainee amounted to €1177.44.

Therapist consultations

The price per consultation was determined bottom-up, similar to the training sessions. The attending therapist was assumed to be a health psychologist and therefore calculated salary costs as before were used (€ 44.97 per hour). Multiplied by 44% overhead, housing and depreciation costs [[1](#_ENREF_1)], the price per hour of consultation amounted to €64.76. In SWORD, there was a total of 6.75 hours of therapist contact involved.

| **Component** | **Total annual costs (€)** | **Costs per patient (€)** |
| --- | --- | --- |
| Website development and update costs | 5,695 | 10 |
| Psychologist training & supervision | 2,590 | 4 |
| User licences | 8,712 | 15 |
| Therapist consultations |  | 437 |
| Total intervention costs |  | **466** |

**Appendix 3.** Mean CWS scores, EQ-5D utility scores and EORTC QLQ-C30 utility scores over time by study group. Error bars represent 95% confidence intervals. Abbreviations: CAU, care as usual; bCBT, blended cognitive behavior therapy; CWS, Cancer Worry Scale; EQ-5D, EuroQol five dimensions questionnaire; QLQ-C30, European Organisation for Research and Treatment of Cancer Quality of Life Questionnaire C30.

**Appendix 4.** Results of the different sensitivity analyses of the primary outcome (CWS).

|  |  | **bCBT** | |  | **CAU** | |  |  |  |
| --- | --- | --- | --- | --- | --- | --- | --- | --- | --- |
| **Analysis** | Measurement | Mean | SE/SD |  | Mean | SE/SD | Mean difference | 95% CI | p |
|  |  |  |  |  |  |  |  |  |  |
| Recurrences excluded |  | n=37 | |  | n=41 | |  |  |  |
|  | T0 | 19.297 | 0.544 |  | 19.756 | 0.579 |  |  |  |
|  | T1 | 14.501 | 0.468 |  | 18.275 | 0.455 | -3.774 | -5.056 to -2.492 | 0.000 |
|  | T2 | 13.477 | 0.504 |  | 17.895 | 0.515 | -4.418 | -5.836 to -3.001 | 0.000 |
|  | T3 | 14.232 | 0.507 |  | 16.464 | 0.523 | -2.232 | -3.665 to -0.799 | 0.002 |
|  |  |  |  |  |  |  |  |  |  |
| Per protocol |  | n=23 | |  | n=30 | |  |  |  |
|  | T0 | 18.435 | 0.576 |  | 19.600 | 0.689 |  |  |  |
|  | T1 | 14.290 | 0.542 |  | 17.544 | 0.472 | -3.253 | -4.685 to -1.823 | 0.000 |
|  | T2 | 13.290 | 0.621 |  | 17.377 | 0.542 | -4.087 | -5.722 to -2.451 | 0.000 |
|  | T3 | 14.247 | 0.619 |  | 16.044 | 0.540 | -1.797 | -3.426 to -0.168 | 0.031 |

NOTE. In the first sensitivity analysis, participants who reported a recurrence were excluded from the analysis dataset. The per protocol sensitivity analysis included participants who completed the bCBT intervention (intervention arm) and who had full follow-up data on FCR.

Abbreviations: bCBT, blended cognitive behaviour therapy; CAU, care as usual.

**Appendix 5.** Clinically significant improvement in FCR severity and Self-rated improvement at 9 months and 15 months follow-up by treatment group for complete cases.

|  | **bCBT** | | |  | **CAU** | | |  |
| --- | --- | --- | --- | --- | --- | --- | --- | --- |
|  | total n | No. | % |  | total n | No. | % | Fisher’s Exact Test P |
| **Reliable change, improvement*** |  |  |  |  |  |  |  |  |
| T2 | 37 | 29 | 78 |  | 32 | 6 | 19 | 0.000 |
| T3 | 32 | 17 | 53 |  | 30 | 10 | 33 | 0.133 |
| **Clinically Significant Change**** |  |  |  |  |  |  |  |  |
| T2 | 37 | 21 | 57 |  | 32 | 3 | 9 | 0.000 |
| T3 | 32 | 16 | 50 |  | 30 | 7 | 16 | 0.038 |
| **Clinically Significant improvement***** |  |  |  |  |  |  |  |  |
| T2 | 37 | 19 | 51 |  | 32 | 1 | 3 | 0.000 |
| T3 | 32 | 11 | 34 |  | 30 | 5 | 17 | 0.150 |
| **Self-rated improvement†** |  |  |  |  |  |  |  |  |
| T2 | 38 | 28 | 74 |  | 32 | 13 | 41 | 0.007 |
| T3 | 32 | 23 | 72 |  | 30 | 12 | 40 | 0.020 |

Abbreviations: CAU, care as usual; bCBT, blended cognitive behavior therapy.

* Statistically reliable change, reliable change index <-1.96.

** Decrease of the Cancer Worry Scale score to the low fearful range (<14).

*** Criteria of reliable improvement and clinically significant change are both met.

† Self-rated improvement had occurred if the patient answered “yes” to the statement, “I have completely recovered from FCR” or “I feel much better but still experience some symptoms of FCR.”

**Appendix 6.** Mean costs per assessment per resource category for complete cases.

|  | **CAU** | | | | | |  | **bCBT** | | | | | | | |
| --- | --- | --- | --- | --- | --- | --- | --- | --- | --- | --- | --- | --- | --- | --- | --- |
|  | **T1 (n=33)** | | **T2 (n=21)** | | **T3 (n=19)** | |  | **T1 (n=34)** | | **T2 (n=32)** | | | **T3 (n=22)** | | |
| **Resource category** | Mean | SD | Mean | SD | Mean | SD |  | Mean | SD | Mean | SD | Mean | | SD | |
|  |  |  |  |  |  |  |  |  |  |  |  |  | |  | |
| Medical specialist | 151 | 157 | 205 | 211 | 166 | 185 |  | 82 | 82 | 154 | 131 | | 210 | | 189 |
| General practitioner | 32 | 35 | 46 | 42 | 31 | 26 |  | 18 | 27 | 35 | 50 | | 41 | | 54 |
| Psychosocial help | 72 | 214 | 176 | 268 | 205 | 602 |  | 3 | 17 | 5 | 19 | | 5 | | 16 |
| Paramedical help | 87 | 198 | 146 | 376 | 134 | 461 |  | 82 | 191 | 191 | 399 | | 174 | | 301 |
| Diagnostics | 127 | 260 | 162 | 142 | 92 | 112 |  | 48 | 86 | 103 | 149 | | 113 | | 151 |
| In-hospital days | 121 | 486 | 691 | 1523 | 271 | 924 |  | 102 | 462 | 64 | 248 | | 0 | | 0 |
| Home care | 110 | 476 | 288 | 1124 | 32 | 138 |  | 0 | 0 | 158 | 807 | | 0 | | 0 |
| Medication | 57 | 85 | 81 | 123 | 54 | 107 |  | 133 | 586 | 248 | 1155 | | 325 | | 1394 |
| Aids and appliances at home | 78 | 197 | 95 | 164 | 68 | 188 |  | 92 | 214 | 90 | 256 | | 137 | | 310 |
| Alternative medicine | 11 | 63 | 20 | 79 | 0 | 0 |  | 14 | 72 | 20 | 67 | 5 | | 26 | |
| **Medical costs subtotal** | 844 | 1196 | 1909 | 2332 | 1051 | 1415 |  | 573 | 803 | 1068 | 1597 | 1009 | | 1399 | |
|  |  |  |  |  |  |  |  |  |  |  |  |  | |  | |
| Informal care | 153 | 394 | 266 | 688 | 212 | 623 |  | 110 | 360 | 297 | 793 | 99 | | 279 | |
| Absenteeism from paid work | 422 | 1722 | 1003 | 2932 | 161 | 438 |  | 306 | 1109 | 135 | 511 | 1014 | | 4754 | |
| **Non-medical costs subtotal** | 575 | 2023 | 1268 | 2947 | 373 | 754 |  | 415 | 1141 | 431 | 899 | 1113 | | 4979 | |
|  |  |  |  |  |  |  |  |  |  |  |  |  | |  | |
| Gross total costs | 1419 | 2738 | 3178 | 4397 | 1424 | 1997 |  | 989 | 1382 | 1500 | 1945 | 2122 | | 5187 | |
| Gross total costs (imputed) | 1353 | 2426 | 2849 | 3261 | 1962 | 2073 |  | 1008 | 1260 | 1731 | 1893 | 2795 | | 4161 | |
| **Incremental gross total costs** | -631 (95% CI: -2828 to 1600) | | | | | | | | | | | | | | |
|  |  |  |  |  |  |  |  |  | | | | | | | |
| Intervention costs |  |  |  |  |  |  |  | 466 | | | | | | | |
| Net total costs T0-T3* | 6165 (95% CI: 4657 to 7850) | | | | | |  | 6001 (95% CI: 4552 to 7664) | | | | | | | |
| **Incremental net total costs*** | -164 (95% CI: -2502 to 2018) | | | | | | | | | | | | | | |

NOTE. Complete cases are participants who completed and returned the cost diary for the given period. Gross total costs consisted of combined medical and non-medical costs. Missing gross total cost data at each assessment were imputed and bCBT Intervention costs were added to obtain the Net total costs.

Abbreviations: bCBT, blended cognitive behaviour therapy; CAU, care as usual; SD, standard deviation; CI, confidence interval; T1, period between baseline and three months post-baseline assessment; T2, period between three months post-baseline and nine months post-baseline assessment; T3, period between nine-months post-baseline and fifteen months post-baseline assessment.

*Net total costs and Incremental net total costs do not exactly follow from Gross total costs and Intervention costs since the Net total costs were 1000x bootstrapped in order to obtain the 95% confidence intervals.

**References**

1. Richtlijn voor het uitvoeren van economische evaluaties in de gezondheidszorg - Guideline for conducting economic evaluations in healthcare. Zorginstituut Nederland 2016.

2. Nederlandse Zorgautoriteit. Tarievenlijst eerstelijnsdiagnostiek - Price list for first line diagnostics. <http://www.nza.nl/regelgeving/bijlagen/Bijlage_1_bij_TB_CU_7078_01_Tarievenlijst_Eerstelijnsdiagnostiek>. 2014. <http://www.nza.nl/regelgeving/bijlagen/Bijlage_1_bij_TB_CU_7078_01_Tarievenlijst_Eerstelijnsdiagnostiek>.

3. Zorginstituut Nederland. Medicijnkosten - Medication costs. https://[www.medicijnkosten.nl/](http://www.medicijnkosten.nl/). 2017. https://[www.medicijnkosten.nl/](http://www.medicijnkosten.nl/).

4. Defined Daily Dose Index. World Health Organization. 2017. https://[www.whocc.no/atc_ddd_index/](http://www.whocc.no/atc_ddd_index/).

5. Zorginstituut Nederland. GIPdatabank. https://[www.gipdatabank.nl/](http://www.gipdatabank.nl/). 2017. https://[www.gipdatabank.nl/](http://www.gipdatabank.nl/).

6. Salarisschalen Cao umc 2015-2017, Bijlage A. NFU2015 14-10-2015.
